# Barriers and facilitators for the implementation of co-created nursing innovations in a Dutch university hospital

**DOI:** 10.1097/XEB.0000000000000444

**Published:** 2024-06-27

**Authors:** Myrthe van der Zanden, Onno Helder, Heleen Westland, Erwin Ista

**Affiliations:** 1 Department of Internal Medicine, Section Nursing Science, Erasmus Medical Center, University Medical Center Rotterdam, Rotterdam, Netherlands; 2 Create4Care, Erasmus Medical Center, University Medical Center Rotterdam, Rotterdam, Netherlands; 3 Research Centre Innovations in Care, Rotterdam University of Applied Sciences, Rotterdam, Netherlands; 4 Julius Center for Health Sciences and Primary Care, University Medical Center Utrecht, Utrecht, Netherlands

**Keywords:** delivery of health care, hospitals, implementation science, nursing staff, qualitative research

## Abstract

**Introduction:**

Escalating global nursing shortages require solutions for efficient care, fewer injuries and lost workdays. When commercial solutions are lacking, innovations developed through co-creation can be a viable alternative. However, many co-created nursing innovations are ineffectively implemented. Understanding the barriers and facilitators for the successful implementation of co-created nursing innovations is crucial.

**Objective:**

This study explored nurses’ perceived determinants influencing the implementation of co-created nursing innovations.

**Methods:**

We conducted a qualitative study using semi-structured interviews with nurses at a Dutch university hospital who were working with co-created nursing innovations. The Theoretical Domains Framework (TDF) was employed to identify the barriers and facilitators. Data analysis followed a deductive theoretical thematic analysis approach based on the 14 TDF domains. Determinants were labeled as a barrier, facilitator, or both.

**Results:**

Of the 14 TDF domains, 9 appeared relevant, including Intention, Professional Role and Identity, and Knowledge. The absence of a “superuser,” lack of storage space, and negative experiences during use were perceived as barriers to the implementation of nursing innovations. The importance of time-saving features, user-friendliness, reminders to use the innovation, and team enthusiasm were perceived as facilitators.

**Conclusions:**

Participants confirmed that the Social Influences domain, combined with Behavioral Regulation and Knowledge, were barriers to implementation, while Intentions and Reinforcements and Rewards were seen as facilitators.

**Spanish abstract:**

**What is already known about the topic**

- Nursing innovations help to reduce injuries and lost workdays for nurses.
- Nursing innovations are often ineffectively implemented in clinical practice.
- Old habits can hinder the prompt adoption of new innovations.

**What this paper adds**

- Insights into barriers and facilitators perceived by nurses for the implementation of nursing innovations.
- Nurse teams involved in the development of the innovation felt more responsible for the innovation process at the department.
- Co-creation enhances the likelihood of innovations meeting nurses’ needs.

## INTRODUCTION

The shortage of nurses in Europe is expected to increase to 2.3 million by 2030.^1^ The reasons for this shortage include a lack of career support and inadequate workforce planning, insufficient appreciation from managers, and physically demanding work conditions.^2^ Improving the work environment, using technological innovations, and enhancing empowerment and autonomy in staffing decisions are measures that create greater job satisfaction. This, in turn, can make the nursing profession more attractive, potentially reducing shortages.^3^ These factors are not only crucial for retaining nurses today, but also for attracting and retaining future generations of nurses.^4^

Innovations in nursing care are increasingly being implemented,^5^ aimed at reducing physical workload (e.g., heavy tasks such as lifting patients or moving beds) and promoting ergonomic practices. Several reports indicate that multifaceted ergonomic health care innovations and programs have resulted in a 20% to 40% of reduction in injuries and lost workdays for nurses.^6^

Nursing innovations can take various forms, from devices such as smart beds and portable monitors, new products and tests, to new forms of health professional training. When commercial solutions for unresolved daily issues are lacking, nurses can develop new innovations though co-creation. This involvement of nurses is also referred as a bottom-up approach.^7^ Co-creation ensures the involvement of end-users in various stages of innovation production, thereby contributing to the acceptance of the innovation.^8^ This can also contribute to nurses’ empowerment, job satisfaction, and autonomy, thereby providing a potential solution to the shortage of nurses.

Many co-created nursing innovations are ineffectively implemented in clinical practice. Reasons include inconsistent use of the innovation, lack of skills to use the innovation effectively, non-user-friendly designs, and insufficient support from colleagues.^9,10^ Such issues can lead to adoption failure.^11^ The first step toward effective implementation is to invite nurses to identify factors that potentially hinder or facilitate successful implementation, as recommended by De Veer *et al.*^12^ To address these potential barriers and facilitators (determinants), the implementation process requires the use of appropriate implementation strategies.^13^ Implementation determinant frameworks are commonly used to identify determinants. These frameworks analyze and categorize factors influencing efforts.^14^ One commonly used framework is the Theoretical Domain Framework (TDF).^15^ This framework comprises 14 domains, enabling an understanding of cognitive, affective, social, and environmental influences on behavior.^16^

The impact of co-created nursing innovations on the reduction of injuries and lost workdays, as well as enhanced empowerment, job satisfaction, and autonomy of nurses during co-creation, highlight the importance of effectively implemented of innovations. Yet, factors influencing the adoption of co-created nursing innovations remain an unexplored area. Therefore, this study aims to explore nurses’ perceptions of barriers and facilitators for the implementation of co-created nursing innovations.

## METHOD

### Design

We applied a generic qualitative research methodology to identify the barriers and facilitators. The TDF informed the data collection and data analysis. The study adhered to the consolidated criteria for reporting qualitative research (COREQ) guidelines.^17^

### Setting and population

Eligible participants were Dutch-speaking nurses from a university hospital in the Netherlands, including nurses, nursing managers, nurse consultants, and nurse trainers. These participants were involved in nursing innovations within the Create4Care health care makerspace^18^ and were working during the implementation of innovations. The Create4Care makerspace is housed within the Erasmus University Medical Center, and develops technical nursing innovations using a bottom-up, co-creation approach with nurses and patients.^19^ These innovations aim to improve patient safety, health care efficiency, and patient comfort.^20^ Examples are shown in Table 1.

**Table 1:**
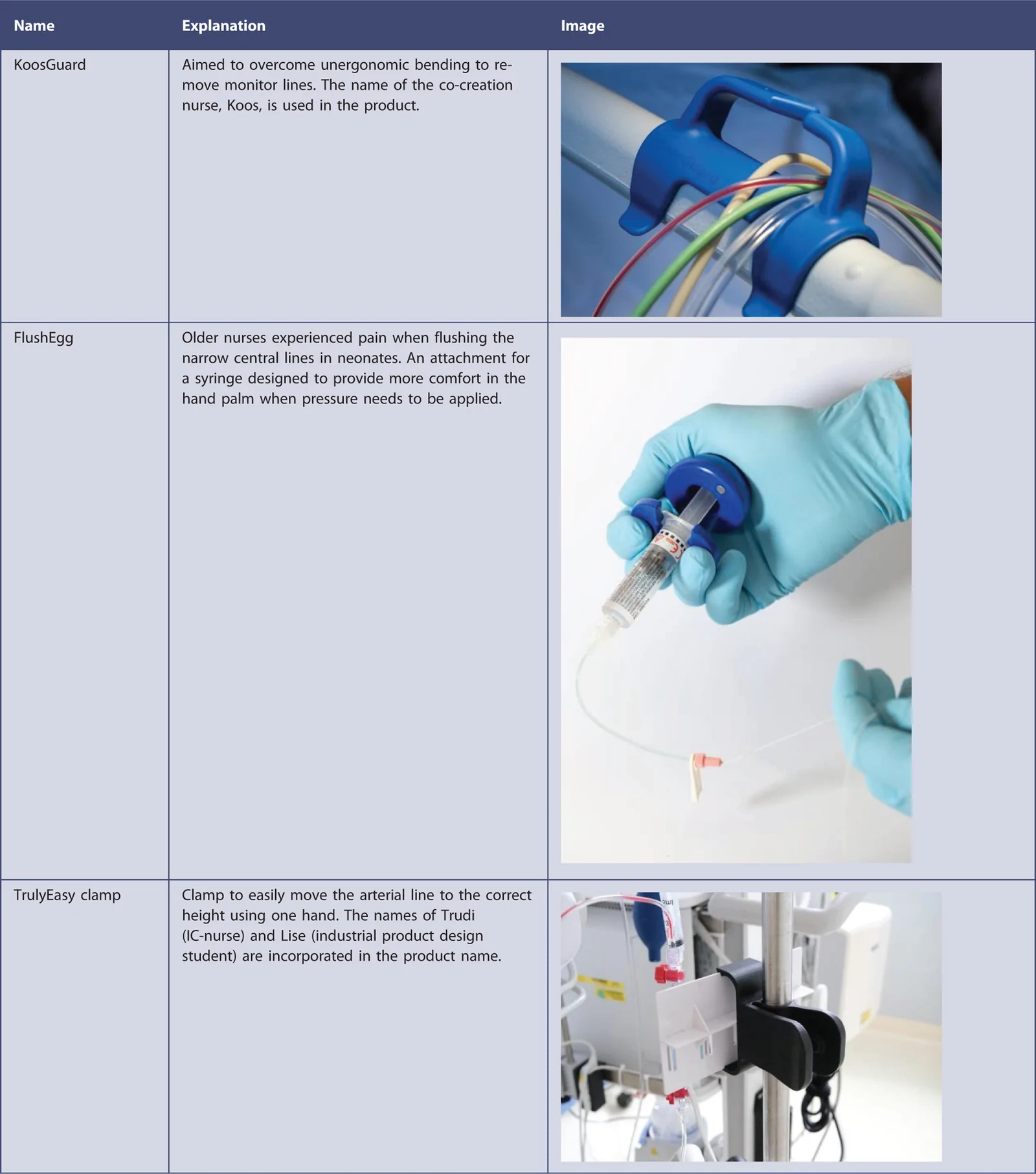
Examples of nursing innovations

**Table 1(Continued):**
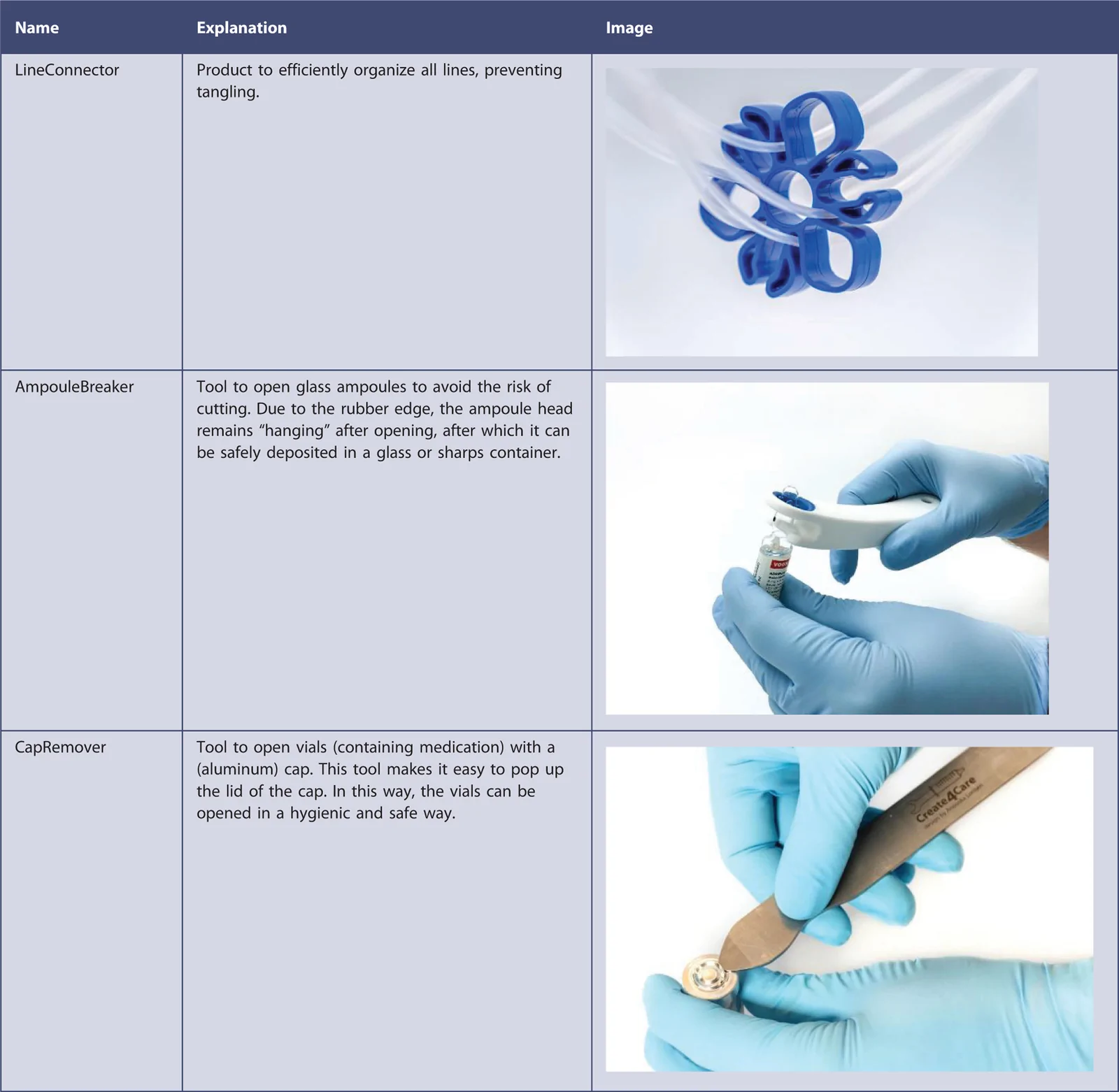
Examples of nursing innovations

### Recruitment and data collection

Purposeful sampling with maximum variation was used.^21^ Co-creators and nurses who use the co-created innovations in the course of their work were contacted by Create4Care [OH] to request permission to share their contact details with the principal researcher [MZ]. Once confirmed, MZ contacted them via email to request participation, in which case an interview was scheduled. Before commencing the interviews, participants provided informed consent and completed a form detailing characteristics such as biological sex, years of work experience, position in the ward, and the co-created innovations they knew and used. Face-to-face interviews were conducted in the hospital by MZ using a semi-structured interview guide. The interview guide was based on the TDF as well as questionnaires and interview guides used in other studies about the implementation of interventions in hospitals.^22,23^ The TDF was used in this study because of its strong theoretical foundation in implementation studies and its comprehensive coverage of potential reasons for implementation issus.^16^ The interview guide included at least one question related to each TDF domain. The opening question of each interview was: “Which co-created innovations are you aware of, and how did you become aware of them?” During the interview, MZ kept observation memos. All interviews were audio-recorded.

### Data analysis

The audio-recordings were transcribed verbatim, pseudonymized, and read and re-read to gain familiarity with the data [MZ]. Theoretical thematic analysis, as described by Braun and Clarke, was used to analyze the data.^24^ The 14 domains of the TDF were used to map the coding. After every two to three interviews, the data were analyzed. Subsequently, the data were coded and discussed within the research team. The first two initial interviews were independently coded and discussed by OH and EI. The codes were then organized into themes and sub-themes; categorized as a barrier, facilitator, or both; and mapped to relevant TDF domains by MZ. A preliminary description of the themes and sub-themes was created using the definitions from the TDF domains.^16^ The data were continually reviewed to verify that the themes and sub-themes were consistent with the transcripts of all interviews [MZ]. The themes and sub-themes were further refined and defined, and quotes were selected [MZ]. NVivo software v. 12 (QRS International, UK) supported the data analysis.

### Study rigor

Data triangulation was employed through interviews with nurses holding different functions, including co-creators and non-co-creators, participants involved during the implementation, and those not involved in the implementation. Researcher triangulation enhanced the study's credibility. To enhance transferability, we provided rich descriptions supported by verbatim quotations. An audit trail documented every step and decision in data collection and analysis for transparency.^25^

### Ethics

The Erasmus MC Medical Review Board reviewed and approved this study as not being subject to the Medical Research Involving Human Subjects Act (MEC-2022-0070). Respondent confidentiality was ensured by pseudonymizing the transcripts and limiting data access to only the research team. The informed consent forms were digitized and securely saved, and all data were stored in a secure environment. The study complies with the Declaration of Helsinki.

## RESULTS

Twenty nurses were invited, and twelve participated in the study, comprising nine nurses, two nurse managers, and one nurse consultant. Nine were co-creators of at least one innovation, and ten were involved in the implementation of the co-created nursing innovations. Two participants were neither co-creators nor directly involved in the implementation of the innovation. However, both had experience using co-created innovations in clinical practice. The data analyses did not reveal any differences in the information gained from the co-creators and non-co-creators. Biological sex was not included in the analysis to examine differences.

Table 2 presents the participants’ characteristics. Interviews were conducted from February to April 2022, ranging from 19 to 45 minutes in length. Data saturation was reached at the eleventh interview, when no new themes or sub-themes emerged, and the twelfth interview confirmed data saturation.

**Table 2:** Characteristics of the participants

Characteristics	Results (%)	Innovations known / mentioned	Results (%)
**Biological sex**		AmpouleBreaker	10 (83)
Male	3 (25)	KoosGuard	8 (66)
Female	9 (75)	LineConnector	6 (50)
**Years of work experience**		Cap Remover	4 (33)
0 – 5 years	0	Flushegg	4 (33)
6 – 10 years	3 (25)	HappyFeed	3 (25)
11 – 15 years	1 (8)	HappyMeasure	3 (25)
16 years or more	8 (66)	Happy breath	3 (25)
**Role – nurses**		Softkees	2 (17)
Nurse	8 (66)	TrulyEasyClamp	2 (17)
Nurse consultant	1 (8)	Dialysis sack elevator	2 (17)
Nurse manager	2 (17)	Cleft lip and palate education model	1 (8)
Nurse – practice supervisor for nurses	1 (8)	NeoNeedleGuard	1 (8)
**Co-creator of one or more innovation(s)**	9 (75)	Syringe puller	1 (8)
**Contributed to implementation**	10 (83)	Nutripress	1 (8)
		Jar viewer	1 (8)

### TDF domains

Of the 14 TDF domains, 9 appeared relevant. No data pertaining to this study were provided for the domains of optimism, emotion, goals, beliefs about consequences, and beliefs about capabilities. Four of the relevant domains related to the co-created nursing innovation and its use, as detailed in Part 1 below, while five domains related to implementation in clinical practice, as detailed in Part 2. Figure 1 summarizes the barriers and facilitators identified by the participants across these domains.

**Figure 1:**
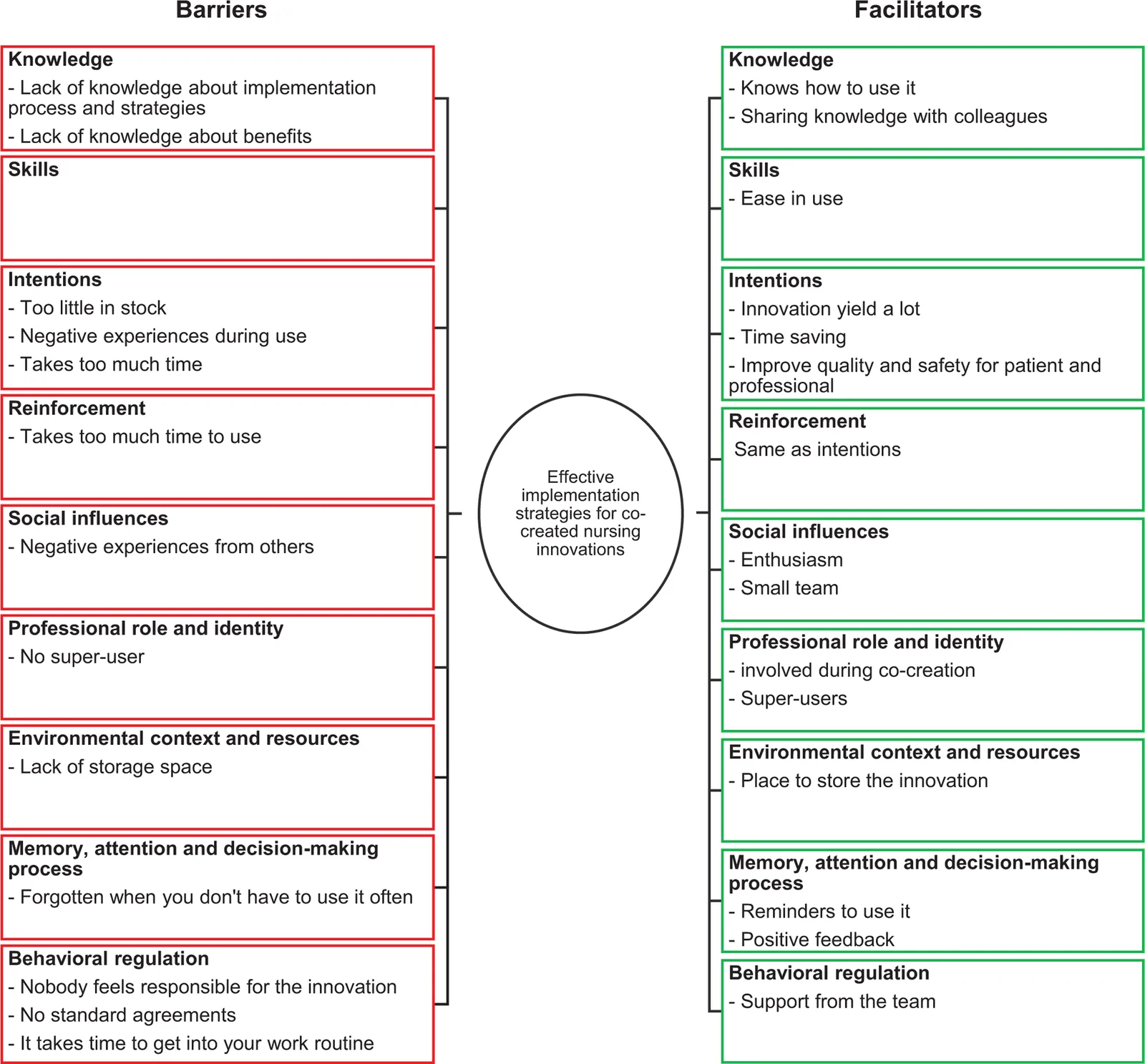
Barriers and facilitators identified by participants.

#### Part 1: Co-created nursing innovations and their use

**Knowledge (An awareness of the existence of something)**

All participants were familiar with at least three co-created nursing innovations within their ward, serving as a facilitators. The initial innovations mentioned, such as AmpouleBreaker, KoosGuard, and CapRemover, were generally not designed for a specific ward or specific end-users, or the participant had a role as co-creator in the innovation they mentioned. Some participants brought up additional co-created innovations that had become integrated into their work routines and were no longer being perceived as innovations.

*“There are many, but because it is always so fragmented in the ward, you are actually not even aware that they are innovations.” (Participant 01)*

Lack of knowledge about correct use can lead to a negative user experience. For example, the AmpouleBreaker can be used in different ways, but only one way is correct. Some participants mentioned that due to negative experiences, they did not use this innovation.

**Skills (An ability or proficiency acquired through practice)**

All participants indicated that they and their colleagues knew how to use the innovations in the ward and perceived that almost all co-created innovations could be used without explanation, which is considered a facilitator.

*“It is such a useful thing and it is very inventive. It is very easy to use.” (Participant 03)*

**Intention (A conscious decision to perform a behavior or a resolve to act in a certain way)**

The intention to use was perceived as a facilitator, but this perception depended on the specific innovation, the user, and social influences. Participants cited various reasons for intending to use an innovation, including positive aspects such its self-explanatory nature, improved efficiency, safety benefits, time-saving aspects, enhanced patient safety, and user comfort. For example, the presence of the TrulyEasy clamp in every IC room made it easily accessible, eliminating time-consuming searches.

*“Sure, yes, because it just looks better, but it really, really makes the job a lot easier.” (Participant 04)*

Participants also expressed the intention not to use a co-created innovation, citing reasons such as when it is not integrated into their work routine, not readily available, or negative past experiences with the innovation. Certain innovations were inadequately stocked, making it more time-consuming to locate them than doing without them. Similarly, if using an innovation took more time than not using it, it was considered an intention not to use. An example mentioned is the LineConnector.

*“If something is complicated and yields little, then you are less inclined to use it.” (Participant 03)*

**Reinforcement and reward (Increasing the probability of a response by arranging a dependent relationship, or contingency, between the response and a given stimulus)**

Two participants co-created innovations which bear their name. They said that having their name associated with the product felt like a reward. Additionally, all participants noted that when an innovation saves time and simplifies processes, they perceive this as a reward and consider it a facilitator.

*“Well, when I do not get any glass in my fingers, and it saves time, that is a reward for me.” (Participant 04)*

#### Part 2: Implementation of co-created nursing innovations in clinical practice

**Social influences (Interpersonal processes that can cause individuals to change their thoughts, feelings, or behaviors)**

Most participants mentioned social support as a facilitating factor during the implementation of an innovation. It was not only the enthusiasm of the co-creators, but also the enthusiasm of their team members that encouraged them to use it. Participants believed that a supportive team was necessary for successful implementation. They noticed that team members involved in decision-making during the implementation process felt partly responsible for the implementation and use of the innovation.

*“I think the key aspect is that the team supports it and that everyone is aware of the problem.” (Participant 07)*

Participants working in small teams of five to ten nurses found this advantageous to motivate their team members and inform them in a more personal manner compared with those in larger teams. Other participants were members of larger teams (over 25 nurses) and often faced challenges in informing every team member about the benefits of the co-created innovation and motivating them to use it.

*“That is the advantage within such a small team that it does not take that much effort to inform everyone” (Participant 07)*

**Professional role and identity (A coherent set of behaviors and personal qualities of an individual in a social or work setting)**

The professional role of the individual participants and the whole team were identified as important facilitators. Co-creators felt more supported by their team members if they had also contributed to the innovation.

*“… the people whom I met in the ward were often immediately enthusiastic about it because they were allowed to think along.” (Participant 03)*

Participants who were not co-creators were more willing to use the innovation when they had provided input during the creation phase.

*“… people still remain positive and therefore also want to use it because they think, ‘I helped with this innovation’.” (Participant 03)*

Most participants noted that having a “superuser” in the ward would help to implement the innovation, as many participants lacked the time and knowledge required. This superuser could either be a colleague from the makerspace or a colleague from the ward who was responsible for implementation.

*“… but actually, translating it into our practice and ensuring that we will also use it. There I notice another gap.” (Participant 03)*

**Environmental context and resources (Any circumstance of a person's situation or environment that discourages or encourages the development of skills and abilities, independence, social competence, and adaptive behavior)**

The environmental context, as perceived by participants, also affects implementation success and was mostly perceived as a barrier. The most common question was: “Where do we store the innovation?” An implementation plan that includes device storage was often lacking, mostly because of a lack of knowledge about implementation.

*“We are very limited in our space, so that was a challenge, where do we store it and put it?” (Participant 07)*

A few participants noted that an innovation is commonly used when it is stored in a fixed place.

*“It is so visible for everyone, so everyone knows it is there.” (Participant 04)*

**Memory, attention, and decision-making (The ability to retain information, focus selectively on aspects of the environment, and choose between two or more alternatives)**

All participants agreed that reminders and fixed places to store the innovation help ensure that they do not forget to use them. Therefore, this domain is also perceived as a facilitator. The mentioned reminders included posters or screensavers, colleagues who encouraged use, along with emails and newsletters.

*“You just have to repeat it frequently. I see you have the IV lines; I’ll get you one of those [KoosGuards]. That's kind of how you have to do it, and at some point, it gets a bit more integrated.” (Participant 08)*

The decision to use co-created innovations such as the CapRemover or AmpouleBreaker depended on the quantity of ampoules to open. Participants used the product only when they had to open multiple ampoules, as this was time-saving.

*“… If I have to open 12 ampoules, for example, then I will do it with the ampoule breaker. But if I only have to do one, I’ll do it without.” (Participant 03)*

**Behavioral regulation (Anything aimed at managing or changing objectively observed or measured actions)**

Habit-breaking was perceived as a barrier to the adoption and use of an innovation. Most participants concurred that once accustomed to working without the innovation, incorporating it into the work routine still requires time, reminders, and a behavioral change.

*“Because you are used to working without it, so you are used to doing without. So, you have to go into a change process to start using it.” (Participant 02)*

## DISCUSSION

We explored the perceived barriers and facilitators for the implementation of co-created nursing innovations, created through a bottom-up, co-creation approach. Our findings indicate that most of the TDF domains act as facilitators, including Skills; Intention; Reinforcement; Social Influence; Memory, Attention, Decision-Making; and Professional Role and Identity. The two domains that where labeled as barriers were Environmental Context and Resources and Behavioral Regulation. The domain of Knowledge was cited as both a barrier and a facilitator. The analyses revealed no difference in the barriers and facilitators experienced during the implementation by co-creators, participants involved in the implementation, and participants who were not co-creators or were not involved in the implementation.

Participants emphasized that a better understanding of the uses and benefits of innovations can contribute to successful implementation, which can be tied back to the domains of Knowledge and Skills. Van Beest *et al.*^26^ confirm this by stating that professionals with a deeper understanding of introduced innovations are more likely to benefit from them and use them. Furthermore, health care professionals are more inclined to use innovations if they recognize and perceive their usefulness.^26^ Therefore, the Intention domain emerged as a facilitator in our study. The use of one of the co-created innovations, the LineConnector, initially required some time, but proved to save time in the longer term. However, the participants had not used the LineConnector long enough to fully experience its benefits. Hence, it is also important for end-users to comprehend the benefits of a co-created innovation and become familiar with it through work experience.

Participants emphasized the importance of the intention to use an innovation and of reinforcement, including rewards, during the implementation process. These findings align with O’Hara *et al.*,^27^ who highlight key factors in implementation, including the quality and quantity of available training for professionals, the availability of rewards for innovation use, sufficient budget for innovations, as well as the quality, accessibility, and user-friendliness of the innovation itself.

Our study demonstrates that during co-creation, the Social Influences and Professional Role and Identity domains positively influence the implementation process—achieved through positive feedback, reminders to use the innovation, assistance during co-creation, support from team members and enthusiasm for the innovation. Various implementation studies have identified Social Influence and Professional Role as positive factors.^28,29^ An innovation champion can contribute to achieving these aspects, according to Birken *et al.*^30^ Professionals with this role can promote implementation of an innovation in four ways: (1) disseminating information; (2) synthesizing information; (3) mediating between strategy and daily routines; and (4) promoting adoption of the innovation.^31^ These professionals may be able to overcome the domains of Environmental Context and Resources, and Behavioral Regulation that were mentioned as barriers. Participants also noted that appointing a “superuser” or innovation champion in the ward would be helpful for the successful implementation of the innovation. Lack of time and lack of knowledge acted as barriers to implementing an innovation, as also acknowledged by Alqahtani *et al.*^32^

Based on the identified barriers and facilitators, tailored implementation strategies can be developed to promote the optimal implementation of nursing innovations. Such strategies could include reminders, ensuring support from the team, and making agreements about the use and storage of the innovation. Innovation champions serve as a valuable learning community and work toward addressing typical implementation issues such as storage, reminders, and disseminating information about the innovation. Future studies should focus on the role innovation champions play in the implementation process. Additionally, further research should focus on gaining a better understanding of the knowledge nurses need to implement an innovation.

### Strengths and limitations

A key strength of this study is the inclusion of perceptions from a variety of nurses, including nurse managers, nurse consultants, co-creators, and nurses who use the innovation, allowing for a deep understanding of the barriers and facilitators to implementation.^33^ Employing a theoretical thematic analysis and the TDF ensured that all relevant constructs related to barriers and facilitators were considered. However, some limitations should be acknowledged. First, this study was single-centered, and no information was obtained about other hospitals or settings. Second, there may be a selection bias, as three-quarters of the participants were co-creators for one or more innovations. These participants may have been more inclined to adopt the innovation and promote it within their teams compared with other professionals.

## CONCLUSION

This study highlighted the barriers and facilitators in the implementation of nursing innovations developed through a bottom-up, co-creation approach. Barriers encountered after implementation included a lack of storage space for the innovations, negative experiences with their use, and unfamiliarity with implementation strategies. Facilitators included rewards for using the innovations, reminders to use the innovations, social support from co-creators and team members, and enthusiasm for the innovation. These findings may inform the development of strategies to improve future implementation endeavors.

## CONSENT FOR PUBLICATION

All authors and respondents have consented to publication.

## AUTHOR CONTRIBUTIONS

MvdZ: Conceptualization, investigation, formal analysis, methodology, writing – original draft, visualization, project administration. OH: conceptualization, validation, writing – review and editing, supervision. HW: writing – review and editing. EI: conceptualization, validation, writing; – review and editing, supervision.

## ACKNOWLEDGMENTS

Ko Hagoort, for editing, and to all the participants.

## Supplementary Material

## Data Availability

The data that support the findings of this study are available upon reasonable request from the corresponding author. The data are not publicly available due to privacy reasons.
