# Supplementary material for: Barriers and facilitators for the implementation of co-created nursing innovations in a Dutch university hospital
Source: JBI Evid Implement. 2024 Jun 27;23(2):190–200. doi: 10.1097/XEB.0000000000000444 (PMC13623065; doi:10.1097/XEB.0000000000000444)
Supplement: Supplementary file 1 [file jbeim-23-190-s001.docx]

Barreras y facilitadores para la implementación de innovaciones co-creadas por enfermería en un hospital universitario holandés

# Resumen

**Introducción**: La creciente escasez de personal de enfermería en todo el mundo exige soluciones para una atención eficiente, menos lesiones y días de trabajo perdidos. Cuando faltan soluciones comerciales, las innovaciones desarrolladas mediante la cocreación pueden ser una alternativa viable, sin embargo, muchas innovaciones co-creadas por enfermería se aplican de forma ineficaz. Es crucial comprender los obstáculos y los factores que facilitan la aplicación con éxito de las co-creaciones innovadoras de enfermería.

**Objetivo**: Este estudio exploró los determinantes percibidos por las enfermeras que influyen en la implementación de innovaciones co-creadas por enfermería.

**Métodos**: Se realizó un estudio cualitativo mediante entrevistas semiestructuradas con enfermeras de un hospital universitario holandés que trabajaban con innovaciones de enfermería co-creadas. Se empleó el Marco de Dominios Teóricos (MDT) para identificar las barreras y los facilitadores. El análisis de los datos siguió un enfoque de análisis temático teórico deductivo basado en los 14 dominios del MDT. Los determinantes se etiquetaron como barreras, facilitadores o ambos.

**Resultados:** De los 14 dominios del MDT, 9 parecieron relevantes, incluyendo la Intención, el Rol Profesional y la identidad, así como el Conocimiento. La ausencia de un «superusuario», la falta de espacio de almacenamiento y las experiencias negativas durante el uso se percibieron como barreras para la aplicación de las innovaciones enfermeras. La importancia del ahorro de tiempo, la facilidad de uso, los recordatorios para utilizar la innovación y el entusiasmo del equipo se percibieron como facilitadores.

**Conclusiones**: Los participantes confirmaron que el dominio de Influencias Sociales, combinado con Regulación Conductual y Conocimiento, fueron barreras para la implementación, mientras que Intenciones, Refuerzos y Recompensas fueron vistos como facilitadores.
